# Health behaviors and problems in Polish homeless men

**DOI:** 10.3389/fpubh.2023.1208877

**Published:** 2023-10-17

**Authors:** Bernadetta Repka, Grzegorz Bejda, Agnieszka Kułak-Bejda, Damian Czarnecki, Marcin Ziółkowski, Anna Mosiołek, Agata Szulc, Napoleon Waszkiewicz, Anna Ślifirczyk, Wojciech Kułak, Elżbieta Krajewska-Kułak

**Affiliations:** ^1^Medical College of the Universal Education Society, Łomża, Poland; ^2^MindHealth Mental Health Center, Białystok, Poland; ^3^The School of Medical Science in Białystok, Białystok, Poland; ^4^Department of Psychiatry, Medical University, Białystok, Poland; ^5^Department of Preventive Nursing, Nicolaus Copernicus University, Toruń, Poland; ^6^Department of Psychiatry, Faculty of Health Sciences, Medical University of Warsaw, Warszawa, Poland; ^7^Siedlce University of Natural Sciences and Humanities, Siedlce, Poland; ^8^Department of Pediatric Rehabilitation and Center of Early Support for Handicapped Children “Give a Chance”, Medical University of Białystok, Białystok, Poland; ^9^Department of Integrated Medical Care, Medical University of Białystok, Białystok, Poland

**Keywords:** homeless, health promoting behaviors, men, mental health, Poland health behaviors, health problems, Poland the homeless

## Abstract

**Introduction:**

Homelessness is considered to be a global problem, independent of the material situation of a given country and occurring in most societies around the world.

**Aim of the study:**

Assessment of the preferred health behaviors of homeless people.

**Materials and methods:**

The study covered 153 men who are homeless and 312 men who are not homeless. The original questionnaire of homeless, and validated the Satisfaction with Life Scale (SWLS), the Health Behavior Inventory (HBI), the General Self-Efficacy Scale (GSES) and the Multidimensional Health Locus of Control (MHLC) Scale were used. The research covered fully completed questionnaires from 153 homeless men staying in Białystok and Gdańsk homelss centers.

**Results:**

On average, the homeless men assessed their health at 6.0 ± 2.7 points, and the non-homeless at 7.8 ± 2.2 points (*p* < 0.001). Significant differences were found between people experiencing a homelessness crisis and the control group in selected aspects concerning the everyday life hygiene of the respondents, health self-assessment, declarations of visits to a specialist and carrying out check-ups, level of satisfaction with life, coping with difficult situations, preferred pro-health behaviors and dimensions of health control. In the used scales, SWLS, HBI, GES, and MHCL, the majority of homeless men obtained average scores. They were rather dissatisfied with their lives, with a low level of effectiveness in coping with difficult situations and obstacles, a low level of health behaviors, and in the scope of health, control increasing the impact of chance.

**Conclusion:**

The level of the presented health behaviors showed statistically significant diversification with all dimensions of the health locus of control, and its internal dimension with age, homelessness phase, the respondents’ criminal history, being under constant medical care, and self-assessment of health.

## Introduction

Homelessness is considered to be a global problem, independent of the material situation of a given country and occurring in most societies around the world. The population of homeless people is internally very diversified, although it is possible to observe in it some life situations typical for this group, most often of a social, economic, psychological, legal or health nature (1). However, the literature on the subject lacks a consensus as to the exact definition of homelessness and a consensus as to whether people living in conditions described as ‘homeless’ consider themselves as such (2). In general, homelessness is defined as ‘the state of a lack of a home’ (3), which does not reflect the actual scope of the problem. Homelessness is living without a home and the inability to satisfy basic needs. Therefore, the definition of homelessness should not exclude people living in inadequate housing conditions or temporary accommodation. The UN report, after Brannon (4), distinguishes the following types of homelessness: rough sleeping (sleeping on the street, under a bridge, or in a public place–temporary, seasonal, short-or long-term); living on the sidewalk (use of the ‘pitch’ and shelter under cardboard, fabric or plastic–short- or medium-term); squatting (regularly staying in the same abandoned building for a short or medium period); living in poor, often unsafe accommodation (on boats or other floating platforms without protection or services, which do not pass all adequacy tests–long-term or permanent) and refugee camps (life without the possibility of returning home–long-term or permanent). Based on ETHEOS 2008, the Polish Typology of Homelessness was developed (5, 6); it distinguishes rooflessness (living without a roof over their head in public spaces), houselessness (staying in facilities for the homeless, in shelters, temporary and transitional accommodation, medical and penitentiary institutions without a residential address); unsecured accommodation (living in unsecured/insecure accommodation temporarily with family/friends); inappropriate/inadequate accommodation (living in temporary/unconventional, fragile constructions/structures – mobile homes, unconventional buildings, temporary structures).

Nearly 155 million people are thought to be homeless and stay in temporary shelters, refugee camps, and other transitional conditions, with another billion living without adequate shelter. It is estimated that by 2050, this number will reach 3 billion. The largest increase in the homelessness rate was recorded in Iceland (by 168% from 2009 to 2017, although the homelessness rate remained low, at 0.1% of the total population). In turn, Norway and Finland recorded the largest decreases in the homelessness rate (in Norway, it fell by 40% in 2012–2016, and in Finland by 39% in 2010–2018). In such countries as Austria, Canada, Denmark, Poland, Slovenia, and Sweden, the homelessness rate has remained relatively stable in recent years (7). In Israel, in 2018–2020, the homeless population almost doubled, from about one person to almost 800 people (7).

At this point, it is worth noting that compared to the countries of Western Europe and other developed countries of the world, where the issue of homelessness has been the subject of systematic reflection in social sciences for many decades, this phenomenon was scientifically diagnosed in Poland very late (8) and it is a problem that is still not fully diagnosed. A relatively new phenomenon in Poland is the increase in the group of homeless foreigners, who often stay illegally, and the so-called ‘homeless by choice’ who wander from city to city (free birds, ‘giants’, vagabonds), who reject all applicable norms and consciously remain on the margins of social life (1).

Homelessness is associated with poorer health status and affects men, women, children, and veterans (9).

Several recent studies indicated that 20–31% of homeless adults endorse such alcohol related problems (10, 11). Compared with housed populations, alcoholism, anemia, and growth problems are more common among homeless persons.

In this study, we tried to evaluate the health behaviors in a group of men experiencing a homelessness crisis in Poland.

Health behaviors have been defined as ‘overt behavioral patterns, actions, and habits that relate to health maintenance, to health restoration, and to health improvement’ (12). A variety of behaviors fall within such a definition, including smoking, alcohol use, diet, physical activity, sexual behaviors, physician visits, medication adherence, screening, and vaccination. Four widely studied health behaviors that are a regular focus of attention are smoking, binge drinking and physical activity (13, 14).

Many health psychology research has examined the psychological determinants of health behaviors (15). Several general models of such determinants have been developed including the health belief model; protection motivation theory, self-determination theory, theory of reasoned action/theory of planned behavior; and social cognitive theory. These models contain several common determinants: intentions, self-efficacy, outcome expectancies, perceived susceptibility, and perceived severity.

Medical anthropology studies how health and illness are shaped, experienced, and understood in the context of cultural, historical, and political forces. Perhaps anthropology’s greatest contribution to our knowledge of homelessness has been a description and understanding of the methods of adaptation and survival in life on the streets and in the shelters (16). The thick, ethnographic descriptions of the daily rounds of the homeless have brought the concept of “the street” to life in these studies. As a group, anthropologists see the street (in its full metaphoric sense) as one of the sites for the kinds of adaptations to contemporary life that some homeless people make.

Understanding the experiences, practices, and perceptions of homeless people in health behaviors is essential to perform effective interventions to improve health outcomes. Furthermore, there are few studies on health behaviors in homeless people.

We wanted to explore selected aspects of mental health including health behaviors, self-efficacy, and locus of control in homeless men. The detailed aims included the assessment of selected health behaviors, need for health care and education, life satisfaction, the strength of the general conviction of the respondents as to the effectiveness of coping with difficult situations and obstacles, sense of the health locus of control and the dependence of the above on the phase of homelessness in the group of people experiencing homelessness crisis compared to those who do not experience it.

## Materials and methods

The research performance received the consent of the Bioethics Committee, R-I- 449/2013. The main research was preceded by a pilot study in each group of 50 people, which made it possible to verify the clarity of the statements formulated in the questionnaires and to finally develop the original questionnaire. The research covered fully completed questionnaires from 153 homeless men staying in Białystok at the St. Brother Albert Home for the homeless of Caritas of the Archdiocese of Białystok, Men’s Night Shelter of Caritas of the Archdiocese of Białystok, Women’s Night Shelter of Caritas of the Archdiocese of Białystok, Emergency Help Point of the ELEOS Orthodox Mercy Center of the Białystok-Gdańsk Diocese and 312 people who do not experience a homelessness crisis (control group). The selection of the groups was purposeful, and the research was carried out with the diagnostic survey method with the use of:

### Instruments

The original questionnaires (version for the homeless and those who are not homeless) consisting of 40 particular questions and questions concerning various aspects of homelessness was used. This survey was not validated. It consists of three parts.

PART I

1. Gender.
2. Age.
3. Marital status.
4. Do you have any children?
5. If so, do you keep in touch with them?
6. Last place of permanent residence:
7. Last place of work.
8. Education.
9. Have you ever had a criminal record?
10. Do you have an ID card?
11. Do you have permanent registration?
12. What are your current sources of income?
13. Are you ready to start work in the coming days if possible?
14. Do you think it is possible to get out of homelessness?

PART II

1. What phase of homelessness are you in?
2. Why did you become homeless?
3. Please indicate where you have been staying in the last year.
4. Do you have health insurance?
5. Do you have a certified disability group or degree of disability?
6. Do you use social assistance?
7. What forms of social assistance do you use?
8. What social assistance facilities do you know for the homeless?

PART III

1. On a scale of 0 to 10, how would you rate your current health?
2. When were you last hospitalized, and why?
3. How often do you use medical care?
4. Are you under constant medical supervision?
5. How often did you use medical services during the last year?
6. Do you follow the doctor’s recommendations?
7. What do you usually do when you feel sick, in pain, or have any ailments?
8. What medications have you used in the last year?*.
9. What are the most important diseases that currently affect you?
10. How often do you have a bath during the week?
11. When you last visited physician or dentist, he had an x-ray of the lungs.
12. How often do you eat the following meals - first breakfast, second breakfast, lunch, afternoon tea, dinner.
13. Do you smoke cigarettes? If so, since when, how much per day, how often.
14. Do you drink alcohol? If so, since when, how much per day, how often.
15. Which ailments do you feel Headaches, Lumbar spine pain, Cervical spine pain, Abdominal pain, Pain in lower limbs, Physical fatigue, Mental fatigue, Susceptibility to stress, Insomnia, Other, what.
16. Do you have any difficulties in performing the following daily activities, such as washing, dressing, moving around, preparing meals.
17. Do you have access to health services?
18. Where is access to health services for the homeless hindered?
19. Which doctor do you have the most difficult access to?

The standardized Satisfaction with Life Scale (SWLS), *Diener, Emmons, Larsen, Griffin, in Polish Juczyński,* version for other professionals who are not psychologists, to assess the extent to which the respondent relates to their current life was used (17). The Satisfaction With Life Scale (SWLS) consists of five statements. The subjects assessed the degree to which the statements describe their lives so far: 1 meant “I strongly disagree,” 2 – “I disagree,” 3 – “I somewhat disagree,” 4 – “I neither agree nor disagree,” 5 – “I somewhat agree,” 6 – “I agree” and 7 – “I strongly agree.” The scores were summed up, and the general result described the level of satisfaction with one’s life. The scores could range from 5 to 35 points, where higher values corresponded to higher satisfaction with life: 5–9 points – for a person extremely dissatisfied with his life, 10–14 points – for a person dissatisfied with his life, 15–19 points – a person slightly dissatisfied with his life, 20 points – a person neither satisfied nor dissatisfied with his life, 21–25 points – a person slightly satisfied with his life, 26–30 points – a person satisfied with his life, 31–35 points – a person delighted with his life. In the interpretation of the results, the specificity of the sten scale was also taken into account. 1–4 sten scores were considered low, 7–10 sten scores were considered high, and 5–6 sten scores were considered average. The Cronbach’s α values start at 0.86 (15).

The standardized Health Behavior Inventory (HBI) according to Juczyński – containing 24 questions describing various types of health-related behaviors (eating habits, preventive behaviors, positive mental attitudes, health practices) in the last 12 months (17). The answers to these questions were graded using a five-point scale where one corresponds to nearly never and five to nearly always. Based on this point system, the mean level of health behaviors was calculated for each category. The total HBI was calculated as the sum of all points gathered. Theoretically, the total HBI could range from 24 points (all answers graded 1) to 120 points (all answers graded 5). These values are presented and interpreted using the standard 10 scale (women: low 24–77 pts., moderate 78–91 pts., high 92–120 pts.; males: low 24–71 pts., moderate 72–86 pts., high 87–120 pts). Cronbach’s reliability index (alpha index) of HBI was calculated to be 0.85, ranging from 0.60 to 0.65 depending on the analyzed category of health behaviors.

The General Self-Efficacy Scale (GSES)–R. Schwarzer, M. Jerusalern, Z. Juczyński–measuring the strength of an individual’s general conviction as to the effectiveness of coping with difficult situations and obstacles (17). The respondent chooses one of four possible responses: no −1, rather no - 2, rather yes - 3, yes - 4. The total score presents a general indicator of self-efficacy, which can vary from 10 to 40 points. High scores represent a high sense of self-efficacy. The general indicator was transformed into standardized units and was interpreted according to the characteristics of the sten score. 1–4 sten scores were considered low, 7–10 sten scores were considered high, and 5–6 sten scores were considered average. The Cronbach’s α of the scale is high – 0.85.

The Multidimensional Health Locus of Control (MHLC-B) Scale version B of B. Wallston, B. S. Wallston, R. DeVellis, in the Polish adaptation of Juczyński – assessing generalized expectations of the respondents in three dimensions of the health locus of control: internal (conviction that control over my own health depends on me); the impact of others (conviction that the state of my health is the result of the impact of others, mainly medical personnel) and chance (the state of health depends on chance or other external factors). The results obtained allow the classification of the respondents into the following types: Strong Internal, Strong External, Decreasing the Impact of Others; Increasing the Impact of Others; Decreasing the Impact of Chance; Increasing the Impact of Chance, Undifferentiated-Strong and Undifferentiated-Weak (17).

### Statistical analysis

The Statistica 13.0 PL program was used for statistical calculations. The Chi2 test was used to compare response rates between groups. Statistical relationships between satisfaction with life, health behaviors, coping with difficult situations and dimensions of the health locus of control in the surveyed group of homeless men and age, phase of homelessness and criminal record calculated with the use of multiple regression analysis. Statistical significance was evaluated at *p* < 0.05.

### Characteristics of the study group

In the study group of 153 homeless men, the largest number was in the age range from 51 to 60 (36.6%), divorced men (55.5%), having children (59.5%), but not maintaining contact with them (53.6%). Most frequently, the respondents had a permanent address of residence in a city with a population over 200,000 (59.6%) and vocational education (52.3%), Details are shown in Table 1.

**Table 1:** Characteristics of the study group.

	Number of men/percentage			
Data	Homeless *n* = 153		Not homeless *N* = 312	
Age				
<20 years old	3	1.9%	0	0%
21–30 years old	13	8.5%	58	18.6%
31–40 years old	28	18.3%	92	29.5%
41–50 years old	25	16.3%	64	20.5%
51–60 years old	56	36.6%	72	23.1%
61–70 years old	25	16.3%	14	4.5%
>70 years old	3	1.3%	12	3.8%
Permanent residential address				
City with a population over 200,000	91	59.6%	140	44.9%
City with a population of 50,000-200,000	30	19.6%	50	16.0%
City with a population below 50,000	16	10.5%	58	18.6%
Rural	13	8.5%	64	20.5%
Education				
Master’s degree	10	6.5%	116	37.2
Bachelor’s degree	0	0	92	29.5%
Secondary education	48	31.4%	58	18.6%
Vocational education	80	52.3%	46	14.7%
Primary education	3	1.9%	0	0
No data	12	7.8%	0	0
Marital status				
Married	17	11.1%	162	51.9%
Widower	13	8.5%	62	19.9%
Single	49	32.0%	58	18.6%
Divorced	68	55.5%	18	5.8%
Separated	3	2.0%	12	3.8%
No data	3	2.0%	0	0%
With children				
Yes	91	59.5%	240	76.9%
Maintaining contact	71	78.0%	240	76.9%
Lack of contact	20	22.0%	0	0%
No	62	40.5%	72	23.1%
Last employment				
Job in a state-owned company	79	51.6%	200	64.1%
Job in a private company	29	19.0%	112	35.9%
Person who has never worked	1	0.7%	0	0
Source of income				
No income	38	24.8%	0	0%
Social benefit	54	35.3%	0	0%
Disability pension	3	3.9%	38	12.2%
Pension	6	3.9%	40	12.8%
Begging	3	2.0%	0	0%
Gathering	9	5.9%	0	0%
Undeclared work	14	9.2%	0	0%
Intervention works	8	5.3%	0	0%
Occasional work	1	0.7%	0	0%
Recycling	8	5.3%	0	0%
Family help	6	3.9%	60	19.2%
Full-time job	0	0	122	39.1%
Scholarship	0	0	50	16%
Criminal record				
No	75	49.1%	0	0
Yes	78	50.9%	312	100%
Having an ID card				
No	19	12.4%	0	0
Yes	134	87.6%	312	100%
Having a permanent address				
Yes	36	23.5%	312	100%
No	109	71.2%	0	0
Once yes	8	5.3%	0	0

## Results

The most common reasons for homelessness were eviction, residence address deregistration (41.8%), family conflicts (30.7%), unemployment, lack of work and lack of sources of income (22.2). Details are shown in Table 2.

**Table 2:** Selected aspects of homelessness in the group of respondents.

Problem	Number of people *N* = 153		Problem	Number of people *N* = 153	
Causes of homelessness*			Place of last residence*		
Eviction, deregistration	64	41.8%	Shelter	38	24.8%
Family conflicts	47	30.7%	Night shelter	45	29.4%
Alcohol	5	3.3%	Warming center	53	34.6%
Gambling	7	4.6%	Staircase	12	7.8%
Domestic violence	5	3.3%	Gazebos, allotment buildings	17	11.1%
Debt	4	2.6%	Basements	6	3.9%
Unemployment, lack of work or other sources of income	34	22.2%	Being put up by family or friends	16	10.5%
Leaving prison	7	4.6%	Attics	4	2.6%
Poor health status	5	3.3%	Park	5	3.3%
Conflicts due to a lack of social tolerance	8	5.2%	Stations, wagons, railway sidings	1	0.7%
Disability	4	2.6%	Heating pipes and nodes	1	0.7%
Own free choice	9	5.6%	Caritas facility	8	5.2%
Nervous breakdown	1	0.7%	Vacant buildings	6	3.9%
Divorce	3	2.0%	Forest	1	0.7%
Depression	1	0.7%	Penal institution	2	1.4%
Apartment fire and lack of a substitute apartment	1	0.7%	Center helping to leave homelessness	2	1.4%
Death of both parents	1	0.7%	Den	7	4.6%
Children moving abroad	1	0.7%	Monar	1	0.7%
Arrest	1	0.7%	No answer	11	7.2%
Sale of an apartment	1	0.7%	Having health insurance		
Helplessness	1	0.7%	Yes	23	15.0%
Home burglary	1	0.7%	No	128	86.7%
Own decision	2	1.4%	Not sure	2	1.4%
Accident at work	2	1.4%	Using social assistance		
No light in the apartment	1	0.7%	Yes	101	66.0%
No water in the apartment	1	0.7%	No	52	34.0%
No data	8	5.2%	Forms of social assistance*		
Phase of homelessness			I do not use it	52	34.0%
0–1	35	22.9%	Financial assistance	81	52.9%
1 to 2 years	9	5.6%	Meal	44	28.8%
2 to 4 years	41	26.8%	Shelter	43	28.1%
4 to 6 years	14	9.2%	Clothes	17	11.1%
6 to 10 years	21	13.7%	Food	18	11.8%
over 10 years	24	15.7%	Food parcels	3	2.0%
No data	9	5.6%	Stay in a shelter	3	2.0%
Is it possible to exit homelessness?			Benefit	1	0.7%
No	11	7.2%			
Yes	114	74.5%			
Hard to say	28	18.3%			

*Multiple answers possible.

The respondents indicated numerous social welfare facilities for the homeless, including night shelters (65.9%), warming centers (44.4%), and other shelters (people – 36.6%).

In the next part of the study, the health habits of the respondents were assessed. 92.2% of the surveyed homeless people and none of the men from the control group did not wash every day. The largest number of the homeless and men from the control group declared that they eat lunch (71.2 and 64.7% respectively) and dinner (77.1 and 58.3% respectively) every day. More than half (55%) of homeless drunk alcohol. Details are shown in Table 3.

**Table 3:** Selected aspects concerning the everyday life hygiene of the respondents.

Problem		Number of men/percentage				*P* value
		Homeless *n* = 153		Not homeless *N* = 312		
Washing/bathing						
Daily washing	No	141	92.2%	0	0%	**<0.001**
	Yes	9	5.9%	312	100%	**<0.001**
	It varies	3	1.9%	0	0%	NS
Frequency of bathing during a week	At all	17	11.1%	0	0%	**<0.001**
	Once	19	12.4%	24	7.7%	NS
	2–3 times	44	28.8%	10	3.2%	**<0.001**
	More than 3 times	2	1.3%	92	29.5%	**<0.001**
	Every day	9	5.9%	146	46.8%	**<0.001**
	It varies	62	40.5%	0	0%	**<0.001**
Eating meals						
1st breakfast	Every day	13	8.5%	164	52.6%	**<0.001**
	Occasionally	109	71.2%	104	33.3%	NS
	At all	31	20.2%	44	14.1%	NS
2nd breakfast	Every day	41	26.8%	114	36.5%	**<0.001**
	Occasionally	31	20.2%	126	40.4%	**<0.001**
	At all	81	53.0%	72	23.1%	NS
Lunch	Every day	109	71.2%	202	58.3%	NS
	Occasionally	22	14.4%	60	19.2%	NS
	At all	22	14.4%	50	16.0%	NS
Afternoon snack	Every day	36	23.5%	80	25.6%	NS
	Occasionally	17	11.1%	100	32.1%	**<0.001**
	At all	100	65.4%	132	42.3%	NS
Dinner	Every day	118	77.1%	182	58.3%	NS
	Occasionally	17	11.1%	72	23.1%	**0.019**
	At all	18	11.8%	58	18.6%	NS
Smoking						
Smoking declaration	No	26	17.0%	14	4.5%	NS
	Now not before yes	9	5.9%	60	19.1%	**<0.001**
	Occasionally	11	7.2%	42	13.5%	NS
	Yes	107	69.9%	196	62.8%	NS
Average smoking period	28.2 ± 12.2; (4 – 50 years)			18.9 ± 6.5; (1–35 years)		
Alcohol consumption						
Alcohol consumption declaration	No	25	16.3%	96	30.8%	**0.012**
	Now not before yes	35	22.9%	12	3.8%	**<0.001**
	Yes	93	60.8%	32	10.3%	**<0.001**
	Do not drink	60	39.2%	108	34.6%	NS
Frequency of alcohol consumption	Every day	33	21.6%	20	6.4%	NS
	Once a week	17	11.1%	24	7.7%	NS
	Several times a month	22	14.4%	66	21.2%	NS
	Several times a year	21	13.7%	94	30.1%	0.003
Daily alcohol consumption	380.3 mL ± 247.2 mL; Min. 100 mL; Max. 660 mL			120.6 mL ± 80.4 mL Min. 25 mL; max. 500 mL		
Type of alcohol	Do not drink	60	39.2%	108	34.6%	NS
	Beer	73	47.7%	50	16.0%	NS
	Wine	25	16.3%	128	41.0%	**<0.001**
	Vodka	35	22.9%	116	37.2%	**0.031**
	Whatever	22	14.4%	0	0%	**<0.001**

Chi-square test. Bold letters for significant values.

The respondents were asked to rate their health from 0 to 10 on a scale. Most frequently, the homeless chose 5 points in their health self-assessment (33 people–21.6%), and men from the control group chose 7 points (23.3%). The homeless men assessed their health poorer (6.0 ± 2.7 points) than the non-homeless (7.8 ± 2.2 points)(*p* < 0.001).

In the homeless group, 98 (64.1%) and 312 (91.0%) in the control group did not have a disability degree certificate.

The respondents declared that:

- they use professional medical assistance when necessary: the homeless–60 (39.2%) and the non-homeless – 80 (25.6%)–*p* = 0.030
- they did not use it at all: the homeless–39 (25.5%) and the non-homeless–10 (3.2%)–(*p* < 0.001)
- several times a year: the homeless–12 (7.8%) and the non-homeless–62 (19.9%)–*p* = 0.005
- several times a month: the homeless–7 people (4.6%) and the non-homeless–32 (10.3%)–NS

In a situation where they feel pain, any ailments:

- they immediately went to the doctor and used medicines prescribed by them–49 (32%) homeless people and 130 (41.7%) men from the NS control group
- they only used their own tested methods – 48 (31.4%) homeless people and 72 (23.1%) men from the NS control group
- they did nothing and tried to wait out the ailments–28 (18.3%) homeless people and 50 (16%) men from the NS control group
- they did not go to the doctor because they could not afford it–25 (16.3%) homeless people and 20 (6.4%) men from the control group *p* = 0.004
- 3 (2%) homeless people and 40 (12.8%) men from the control group had problems with the declaration on this issue *p* < 0.001

21 (13.7%) homeless people and 214 (68.6%) people from the control group (*p* < 0.001) used the help of the general practitioner.

In the last year, 48 (31.4%) surveyed homeless men and 70 (22.4%) from the control group did not use medical assistance–NS.

In the last year, 62 (40.5%) surveyed homeless men and 284 (91%) from the control group were not in hospital–*p* < 0.001.

In the last year they took antibiotics–50 (32.7%) homeless men and 139 (44.6%) from the control group–NS; vitamins–47 (30.7%) homeless men and 176 (56.4%) from the control group–*p* = 0.0019, analgesics–33 (21.6%) homeless men and 11 (3.5%) from the control group–*p* < 0.001; sedatives–31 (20.3%) homeless men and 36 (11.5%) from the control group–*p* = 0.034. No medications were taken by 34 (22.2%) homeless men and 56 (17.9%) from the control group–NS.

During the study, 74 (48.4%) homeless men and 112 (35.9%) from the control group reported they did not suffer from any disorders. Others most often complained about hypertension–14 (9.2%) homeless men and 116 (37.1%) from the control group–*p* < 0.001; back pain–10 (6.5%) homeless men and 18 (5.8%) from the control group–NS, asthma/allergy–8 (5.2%) homeless men and 48 (15.4%) from the control group–*p* = 0.007, heart problems–6 (3.9%) and 34 (10.9%) from the control group *p* = 0.031, diabetes–5 (3.3%) homeless men and 41 from the control group *p* < 0.001, epilepsy, gastric ulcer–4 (2.6%) homeless men alcoholism. In the control group, anemia was also reported–4 (1.3%) people, headaches/migraine–18 (5.8%) people, thyroid diseases–18 (5.8%) people, ophthalmological diseases–21 (6.7%) people, joint degeneration–19 (6.1%) people and lower limb varicose veins–6 (1.9%) people.

The respondents were asked about the declaration of visits for the internist, dentist and lung x-rays. Most often, the homeless claimed that they did not remember the last time they had visited an internist (53.6%), dentist (52.3%) and when they had a lung x-ray (51%). Details are shown in Table 4.

**Table 4:** Declaration of visits to the internist, the dentist, and for a lung X-ray.

		Number of men/percentage				*P*–value
Problem		Homeless *N* = 153		Not homeless *N* = 312		
Last visit to the internist	1–2 months ago	35	22.9%	66	21.2%	NS
	Half a year ago	11	7.2%	84	26.9%	**<0.001**
	1 year ago	9	5.9%	64	20.5%	**<0.001**
	Several years ago	16	10.5%	36	11.5%	NS
	I do not remember	82	53.6%	62	19.9%	NS
Last visit to the dentist	1–2 months ago	16	10.5%	118	37.8%	**<0.001**
	Half a year ago	5	3.3%	94	30.1%	**<0.001**
	1 year ago	23	15.0%	10	3.2%	NS
	Several years ago	29	19.0%	28	9.0%	NS
	I do not remember	80	52.3%	62	19.9%	NS
Last lung X-ray	1–2 months ago	14	9.2%	2	0.6%	**<0.001**
	Half a year ago	9	5.9%	6	1.9%	NS
	1 year ago	18	11.8%	34	10.9%	NS
	Several years ago	34	22.2%	10	3.2%	**<0.001**
	I do not remember	78	51.0%	260	83.3%	**<0.001**

Chi-square test; NS, not significant. Bold letters for significant values.

Homeless men reported, sometimes they have a headache (34%), stomachache (32.7%), physical fatigue (36.6%), mental fatigue (34. 6%), stress (33.3%), insomnia (32%), and, every day, lower back pain (30.7%), and lower limb pain (36.6%). Men from the control group most often had a headache (39.1%), stomachache (31.4%), mental fatigue (33.3%), stress (35.9%), insomnia (42.9%), lower back pain (42.9%), lower limb pain (40.4%), neck pain (37.8%), and, every day, physical fatigue (30.1%). The results are shown in Table 5.

**Table 5:** Declaration of the frequency of experiencing selected ailments.

Health problem		Number of men/percentage				*P*–value
		Homeless *n* = 153		Not homeless *N* = 312		
Headaches	Every day	32	20.9%	52	16.7%	NS
	Once a week	19	12.4%	48	15.4%	NS
	Several times a month	22	14.4%	46	14.7%	NS
	Sometimes	52	34.0%	122	39.1%	NS
	Never	28	18.3%	44	14.1%	NS
Lower back pain	Every day	47	30.7%	40	12.8%	NS
	Once a week	17	11.1%	48	15.4%	NS
	Several times a month	20	13.1%	32	10.3%	NS
	Sometimes	42	27.5%	134	42.9%	**0.033**
	Never	27	17.6%	58	18.6%	NS
Neck pain	Every day	37	24.2%	46	14.7%	NS
	Once a week	17	11.1%	26	8.3%	NS
	Several times a month	19	12.4%	50	16.0%	NS
	Sometimes	36	23.5%	118	37.8%	**0.037**
	Never	44	28.8%	72	23.1%	NS
Stomachache	Every day	29	19.0%	24	7.7%	NS
	Once a week	19	12.4%	14	4.5%	NS
	Several times a month	22	14.4%	42	13.5%	NS
	Sometimes	50	32.7%	98	31.4%	NS
	Never	33	21.6%	52	16.7%	NS
Lower limb pain	Every day	56	36.6%	44	14.1%	NS
	Once a week	15	9.8%	28	9.0%	NS
	Several times a month	19	12.4%	22	7.1%	NS
	Sometimes	40	26.1%	126	40.4%	**0.043**
	Never	23	15.0%	92	29.5%	**0.009**
Physical fatigue	Every day	40	26.1%	94	30.1%	NS
	Once a week	17	11.1%	50	16.0%	NS
	Several times a month	28	18.3%	42	13.5%	NS
	Sometimes	56	36.6%	86	27.6%	NS
	Never	12	7.8%	40	12.8%	NS
Mental fatigue	Every day	35	22.9%	68	21.8%	NS
	Once a week	20	13.1%	26	8.3%	NS
	Several times a month	30	19.6%	60	19.2%	NS
	Sometimes	53	34.6%	104	33.3%	NS
	Never	15	9.8%	54	17.3%	NS
Feeling stressed	Every day	31	20.3%	22	7.1%	NS
	Once a week	20	13.1%	32	10.3%	NS
	Several times a month	25	16.3%	58	18.6%	NS
	Sometimes	51	33.3%	112	35.9%	NS
	Never	26	17.0%	88	28.2%	0.048
Insomnia	Every day	39	25.5%	26	8.3%	NS
	Once a week	19	12.4%	32	10.3%	NS
	Several times a month	25	16.3%	18	5.8%	NS
	Sometimes	49	32.0%	134	42.9%	**<0.001**
	Never	21	13.7%	102	32.7%	**<0.001**

Chi-square test; NS, not significant. Bold letters for significant values.

In the assessment of satisfaction with life (SWLS), the homeless men obtained 15.4 ± 8.1 points on average (people rather dissatisfied with their lives), and in the control group – 23.2 ± 5.5 on average (people rather satisfied with their lives). In assessing the effectiveness of coping with difficult situations and obstacles, the homeless men obtained 18.5 ± 5.7 points, and the control group obtained 29.1 ± 1.2 points. The homeless in the point scale in the assessment of health behaviors (HBI) obtained 62.4 ± 21.9 points on average, and the control group obtained 82.1 ± 10.8 points. The homeless obtained the highest average values for positive mental attitude (2.9 ± 1.1 points on average), and the control group for health practices (3.9 ± 0.7). The homeless and the control group obtained the lowest mean values for proper eating habits (2.3 ± 1.0 and 3.5 ± 0.9, respectively). In assessing the dimensions of the health locus of control, the homeless obtained the highest average values in the dimension of chance – 19.2 ± 9.1 points on average, and the control group in the internal dimension (26.0 ± 5.2). Details are shown in Table 6.

**Table 6:** Results of the analysis using SWLS, GSES, HBI and MHCL-B scales.

	Number of men/percentage				*p*
	Homeless *n* = 153		Not homeless *N* = 312		
SWLS–level of satisfaction with life					
Average values–points	15.4 ± 8.1		23.2 ± 5.5		**<0.001**
Average level of satisfaction with life–sten scores	3.8 ± 2.7		7.1 ± 1.2		**<0.001**
Person definitely dissatisfied with life	39	25.5%	26	8.3%	NS
Person very dissatisfied with life	40	26.1%	12	3.8%	**<0.001**
Person rather dissatisfied with life	24	15.7%	30	9.6%	NS
Person neither satisfied nor dissatisfied with life	7	4.6%	4	1.3%	NS
Person rather satisfied with life	25	16.3%	112	35.9%	**0.001**
Person very satisfied with life	12	7.8%	76	24.4%	**0.005**
Person definitely satisfied with life	6	3.9%	52	16.7%	**<0.001**
GSES–sense of self-efficacy					
Average values–points	18.5 ± 5.7		29.1 ± 1.2		**<0.001**
Average values–sten scores	2.7 ± 1.8		5.3 ± 2.1		**<0.001**
Low sense	129	84.3%	41	13.1%	**<0.001**
Average sense	13	8.5%	55	17.6%	**0.031**
high sense	11	7.2%	216	69.3%	**<0.001**
HBI–Health Behavior Inventory					
Average value of the general HBI indicator	62.4 ± 21.9		82.1 ± 10.8		**<0.001**
Medium sten scores	3.7 ± 2.3		6.4 ± 1.8		**<0.001**
Correct eating habits	2.3.0 ± 1.0		3.5 ± 0.9		**<0.001**
Preventive behaviors	2.7 ± 1.1		3.8 ± 0.8		**<0.001**
Positive mental attitude	2.9 ± 1.1		3.6 ± 0.7		**<0.001**
Health practices	2.6 ± 0.9		3.9 ± 0.7		**<0.001**
Level of health-related behavior					
Low indicator of the intensity of health behaviors	103	67.3%	103	33.0%	NS
High indicator of the intensity of health behaviors	20	13.1%	131	42.0%	**<0.001**
Average indicator of the intensity of health behaviors	30	19.6%	78	25%	NS
MHCL-B–health control dimension					
Internal (I)	17.9 ± 9.9		26.0 ± 5.2		**<0.001**
Impact of others (IO)	17.7 ± 9.7		19.8 ± 4.9		**0.001**
Chance (CH)	19.2 ± 9.1		24.1 ± 6.8		**<0.001**
Strong internal type	2	1.3%	48	15.4%	**<0.001**
Strong external type	15	9.8%	44	14.1%	NS
Decreasing the impact of others type	13	8.5%	20	6.4%	NS
Increasing the impact of others type	9	5.9%	24	7.7%	NS
Decreasing the impact of chance type	5	3.3%	28	9.0%	**0.049**
Increasing the impact of chance type	12	7.8%	40	12.8%	NS
Undifferentiated-strong type	50	32.7%	84	26.9%	NS
Undifferentiated-weak type	47	30.7%	24	7.7%	NS

Chi-square test; NS, not significant. Bold letters for significant values.

Statistically significant correlations were found between the internal dimension of health behavior control and age, homelessness phase, criminal history of the respondents, being under constant medical care and health self-assessment. The ‘Impact of others’ dimension showed significant statistical differences with the level of health behaviors, and the ‘chance’ dimension with the criminal record of the respondents. The level of the presented health behaviors showed statistically significant differences with all dimensions of the health locus of control (Table 7).

**Table 7:** Statistical relationships between satisfaction with life, health behaviors, coping with difficult situations and dimensions of the health locus of control in the surveyed group of the homeless and age, homelessness phase and criminal record.

		SWLS	GSES	HBI	MHCL/DIMENSIONS		
					*I*	IO	CH
SWLS		1.00	0.268	0.669	0.111	0.791	0.622
GSES		0.268	1.00	0.261	0.349	0.303	0.428
HBI		0.669	0.069	1.00	**0.019**	**0.002**	0.261
MHCL/Dimensions	I	0.077	0.244	**<0.001**	1.00	1.00	1.00
	IO	0.367	0.186	**<0.001**	1.00	1.00	1.00
	CH	0.714	0.997	**<0.001**	1.00	1.00	1.00
Age		0.128	0.753	0.370	0.002	0.657	0.054
Phase of homelessness		0.213	0.283	0.160	0.001	0.916	0.347
Criminal record		0.057	0.128	0.116	**<0.001**	0.206	0.005
Being under medical care		0.401	0.512	0.477	**0.004**	0.311	0.751
Health self-assessment		0.139	0.326	0.324	**0.001**	0.714	0.163

Multiple regression analysis. Bold letters for significant values.

## Discussion

In the present study, health behaviors of homeless men showed statistically significant diversification with all dimensions of the health locus of control, and its internal dimension with age, homelessness phase, the respondents’ criminal history, being under constant medical care, and self-assessment of health. We found significant differences between the homeless men and controls in selected aspects concerning the everyday life hygiene, health self-assessment, declarations of visits to a specialist and carrying out check-ups, level of satisfaction with life, coping with difficult situations, preferred pro-health behaviors and dimensions of health control.

In the study by Baranowski (18), which covered 91 homeless people, 80.2% were men and people aged 51 to 60 (37.4%) and 41 to 50 (34.1%). This was confirmed by research conducted by Pindral (7), which also showed a much higher percentage of men in the homeless group (on average four times); research conducted by Śledzianowski (19)–in which he also found the dominance of men (90.7%) and the research from 2019, in which it was shown that the largest number of homeless people were aged 41–60 (45.5% of people) (20). The above was also confirmed in the current study, in which the largest number, i.e., 36.6%, of men were in the age range from 51 to 60.

In the study by Baranowski (18), 46.2% of the homeless declared vocational education, 24.1%–primary or incomplete primary education, 17.6%–secondary education, 7.7%–general secondary education, and 2.2%–lower secondary education or higher education. In the group Pindral [7] studied, 75% of homeless people had post-primary education, a 34% higher indicator than the homeless population. In the study by Śledzianowski (19), 50.8% of homeless people had vocational education, 23.5% had primary education, 21.2% had secondary or incomplete secondary education, and 3.9% had higher education.

The literature data show that the homeless population is also dominated by singles, divorcees, people in separation, children, and wives fleeing from home due to violence by their fathers or husbands (20). The homeless people in the 2011 study in Białystok, Suwałki (21) were most often (44%) divorcees; almost 30% of the respondents were unmarried, 15%–married, and 10% were widowed. This has been confirmed in the current study, where divorced people accounted for 55.5% of all homeless people and singles for 32%.

In the literature on homelessness (22, 23), there are certain levels of isolation of the homeless which make it difficult to get out of homelessness: economic level (failure to meet needs), social level (lack of interpersonal relationships), individual level (emotional disorders, low self-esteem) and institutional level (confusion in institutional support mechanisms). In the research of Baranowski (11), the most frequently indicated factors causing homelessness were family problems (42.9%) and addictions (41.8%). Inhabitants of Łódź studied by Bartczak et al. (22) indicated addictions (86.5%), eviction from the apartment (78.6%), and loss of a job (62.0%) or being abandoned by the closest family (48.1%) as reasons for becoming homeless. According to a 2019 study, the main cause of homelessness was family conflict (32.2%), addiction (28%), eviction, deregistration (26.3%) and relationship breakdown (18.4%) (22). This has also been confirmed in our research because, in the case of our respondents, the homelessness crisis was most often caused by eviction, deregistration (41.8%), and family conflicts (30.7%).

In the research by Baranowski (18), 62.6% of the homeless stayed in various institutions such as hostels, centers for the homeless, or social emergency centers, while the remaining 37.4% occasionally used the offers of eateries and came to various centers for food and clothes. The homeless surveyed by Olech (24), rarely used the services of facilities providing them with shelter, and during the last year, only 7% occasionally lived in a facility for homeless people. According to the 2019 study, 80.2% of the homeless stayed in institutional facilities, such as shelters, while the remaining 19.8% lived outside (20). Contrary to the above data, in the present study, only 24.8% indicated a shelter as the place of their last stay; the respondents most often indicated warming centers (34.6%) and night shelters (29.4%).

In the literature (21, 24, 25), there is a division of the typology of homeless people into homeless by choice, homeless by force, actually homeless at risk of homelessness, homeless by force, temporarily homeless by choice; shallowly homeless deeply homeless and permanently homeless temporarily homeless. Also (26) five stages of homelessness are distinguished: 1. breakdown of the life plan and breakdown of the family; 2. material poverty, cultural poverty, and social poverty; 3. different dimensions of becoming homeless; 4. adaptation to homelessness; 5. actual, chronic homelessness, when full adaptation to the state of homelessness takes place, lasting 6 to 10 years. The largest group in the 2019 study was people experiencing a homelessness crisis for more than 5 to 10 years–(27.8%) and homeless people for up to 2 years (23.4%) (20). In the current study, there were significantly more homeless people in the phase up to years (28.5%) and fewer in the group from 5 to 10 (21.9%). In the study of Baranowski (18) only 8.8% of homeless persons had a permanent legal job,

The main sources of income for the others were: occasional work (15.4% each), social assistance (12.1%), charitable organizations’ assistance (8.8%), disability pension/pension (17.6%), and collecting and selling scrap (5.5%).

Buciński et al. (26) emphasize that the health condition of homeless people is worse than that of the community leading a normal life, and the above depends on many factors and does not allow the treatment of homeless people in a universal way. Basically, the health problem of the homeless is affected by age, length of homelessness, place of stay of the homeless person, aging of the homeless community, and staying in public spaces that increase the risk to health and even life. The issue of homeless people’s health should be of key importance for social policy and the system of helping homeless people.

People without a roof over their heads, more often than others, do not have health insurance or an ID card (27). In the research carried out by Śledzianowski (19), 78.7% of women and 60.3% of men had health insurance. The current studies have also confirmed this, as most (86.7%) of our respondents did not have health insurance.

In the study of Śledzianowski (19), 28.7% had a disability degree certificate, usually moderate (64%). In the group of homeless people from the study conducted in 2011 in Białystok, Suwałki (21), 47% had a moderate disability degree, and 30% had a light disability degree. In contrast to the above, in the current study, 64.1% of homeless people did not have a disability certificate.

Heszen (28) emphasizes that health consists of four main dimensions: mental, social, somatic, and spiritual. In the context of homelessness, every aspect of health is important. 77.4% of homeless people from the Śledzianowski study (19) reported good health. In the current study, the respondents assessed their health as average.

The assessment of the health of the homeless in a shelter in Poznań city (29), showed that the most common diseases they had were parasitic and infectious skin diseases, alcoholism, tuberculosis, hypertension, diseases of the spine and musculoskeletal system, nervous system, and gastrointestinal tract. According to the study (21) conducted in 2011 in Białystok, Suwałki, 50% of the homeless reported feeling unhealthy. In the present study, 48.4% of the homeless claimed they did not suffer from any disorders.

While examining the health of the homeless in a shelter for homeless men in Poznań, Przymeński (29) stated that the homeless usually did not treat their diseases. Śledzianowski (19) demonstrated that 57.1% of the homeless people used medical services last year. In the current study, 31.4% of the respondents did not use medical assistance in the last year.

Przymeński (29) closely connects the poor health of the homeless to their living conditions, including poor hygiene, malnutrition, lack of adequate protection against low temperatures, constant stress, lack of satisfaction with basic mental and emotional needs, and addiction to drugs and alcohol. According to Tędzialgolska et al. (30), the daily functioning of homeless people was hindered by the lack of possibility to care for hygiene due to the lack of intimacy, basic toiletries, access to water, and clean clothes. This is also confirmed by our research, because as many as 92.2% of the respondents did not wash every day.

Błażej and Bartosz (26) think that excessive alcohol consumption is a very special feature of homeless people. In a Polish study (24), about 70–80% of adult homeless men are addicted to alcohol. In a French study (31), one in five homeless people was alcohol-dependent. In the current study, the respondents reported that they most often consume alcohol occasionally (55.5%).

Doctors who care for homeless people notice the following health problems: cardiological problems, the digestive, respiratory, urinary, and hormonal systems, cancers, skin diseases, frostbite, dental deficiencies, and HIV/AIDS (24, 32). In the study by Śledzianowski (19), 16.4% of the homeless suffered from congenital diseases, 3.5% from respiratory diseases, 3.3% from mental and neurological diseases, 2.7% from heart and circulatory system diseases, and 3% from musculoskeletal system diseases. In the current research, the respondents most often complained about hypertension (9.2%), back pain (6.5%), asthma (5.2%), heart problems (3.9%), diabetes, colds, pneumonia (3.3% each), epilepsy, gastric ulcer (2.6% each), alcoholism, prostatic hypertrophy, atherosclerosis, and hernia (2% each).

Homelessness brings such emotional effects as loneliness, powerlessness, fear, and anxiety (26, 33). On the other hand, 63.2% of the homeless people from the Śledzianowski study (19) believed they were valuable, and 61.5% did not lose self-confidence. Homeless people from the study by Bodys-Cupak et al. (34) declared a sense of external control over their health and a low sense of effectiveness. Similarly, in the current study, homeless people showed a low level of effectiveness in coping with difficult situations and obstacles but a high level of internal control, indicating the belief that control over their health lies with them. Unfortunately, the homeless respondents also showed a low level of satisfaction with life and a low level of health behaviors, the highest for the sphere of a positive mental attitude and the lowest for proper eating habits.

In conclusion, it is worth emphasizing once again that the problem of homelessness is a broad and extremely complicated phenomenon (35–37). The struggle for survival and the necessity to satisfy basic needs preclude focusing on one’s health and taking care of its good condition and often contributes to its deterioration. Therefore, several actions should be taken to protect health. Unfortunately, health for people in the homeless crisis is no longer a value and a determinant of action; hence, problems may arise in complying with the basic principles of treatment and in remission diseases. However, in the current study, as many as 60.1% of the respondents claimed that they always follow medical recommendations, 39.2% did it sometimes or never, and 74.5% were not under constant medical care. The above shows that medical assistance for the homeless requires considering several different variables, including the entire social context. Our results suggest that the groups of men whom special measures should cover are those in the homelessness phase up to 4 years and people with a criminal record.

### Study limitations

The small size of the group and the lack of evaluation in the group of women can be considered as limitations of the study.

### Practical implications

Homeless people should be provided with free health care.

Homeless people should have access to specialist doctors.

It is advisable to monitor the health behaviors of the homeless to get to know them better.

### Future research directions

The research should be carried out on a larger population in different country regions.

Research should also include homeless women.

## Conclusion

1. The level of the health behaviors showed statistically significant diversification with all dimensions of the health locus of control and its internal dimension with age, phase of homelessness, criminal history of the respondents, being under constant medical care, and self-assessment of health.
2. Significant differences were found between people experiencing a homelessness crisis and the control group in selected aspects concerning the everyday life hygiene of the respondents, health self-assessment, declarations of visits to a specialist and carrying out check-ups, level of satisfaction with life, coping with difficult situations, preferred pro-health behaviors and dimensions of health control.

## Data availability statement

The raw data supporting the conclusions of this article will be made available by the authors, without under reservation.

## Ethics statement

The studies involving humans were approved by the Bioethics Committee of the Medical University of Bialystok, R-I- 449/2013. The studies were conducted in accordance with the local legislation and institutional requirements. Written informed consent for participation was not required from the participants or the participants’ legal guardians/next of kin in accordance with the national legislation and institutional requirements.

## Author contributions

BR, DC, MZ, AM, AS, and AŚ collected the epidemiological and clinical data. NW processed statistical data. BR, GB, and AK-B drafted the manuscript. BR, GB, and NW revised the final manuscript. WK and EK-K is responsible for summarizing all epidemiological and clinical data. All authors contributed to the article and approved the submitted version.
